# Genetic and Epigenetic Changes in *Arabidopsis thaliana* Exposed to Ultraviolet-C Radiation Stress for 25 Generations

**DOI:** 10.3390/life15030502

**Published:** 2025-03-20

**Authors:** Andres Lopez Virgen, Narendra Singh Yadav, Boseon Byeon, Yaroslav Ilnytskyy, Igor Kovalchuk

**Affiliations:** 1 Department of Biological Sciences, University of Lethbridge, Lethbridge, AB T1K 3M4, Canada; andres.lopezvirgen@uleth.ca (A.L.V.); nsyadava2004@gmail.com (N.S.Y.); slava.ilyntskyy@uleth.ca (Y.I.); 2 Biomedical and Health Informatics, Computer Science Department, State University of New York, 2 S Clinton St, Syracuse, NY 13202, USA; boseon.byeon@oswego.edu

**Keywords:** multigenerational UV-C stress, stress memory, *Arabidopsis thaliana*, intergenerational inheritance, transgenerational inheritance, phenotypic resilience, epigenetic variations, genetic variations, SNPs, INDELs, differentially methylated cytosines, differentially methylated regions

## Abstract

Continuous exposure to stress contributes to species diversity and drives microevolutionary processes. It is still unclear, however, whether epigenetic changes, in the form of epimutations such as, for example, differential DNA methylation, are the pre-requisite to speciation events. We hypothesized that continuous stress exposure would increase epigenetic diversity to a higher extent than genetic diversity. In this work, we have analyzed the effect of 25 consecutive generations of UV-C-stress exposure on the *Arabidopsis thaliana* genome and epigenome. We found no evidence of increased tolerance to UV-C in the progeny of UV-C-stressed plants (F25UV) as compared to the progeny of control plants (F25C). Genetic analysis showed an increased number of single nucleotide polymorphisms (SNPs) and deletions in F25UV plants. Most common SNPs were mutations in cytosines, C to T, C to A, and C to G. Analysis of cytosine methylation showed a significant increase in the percentage of methylated cytosines at CG context in F25UV as compared to F25C or F2C (parental control). The most significant differences between F25UV and either control group were observed in CHG and CHH contexts; the number of hypomethylated cytosines at CHH contexts was over 10 times higher in the F25UC group. F25UV plants clustered separately from other groups in both genomic and epigenomic analyses. GO term analysis of differentially methylated genes revealed enrichments in “DNA or RNA metabolism”, “response to stress”, “response to biotic and abiotic stimulus”, and “signal transduction”. Our work thus demonstrates that continuous exposure to UV-C increases genomic and epigenomic diversity in the progeny, with epigenetic changes occurring in many stress-responsive pathways.

## 1. Introduction

Transgenerational inheritance is defined as the phenomenon where traits are passed from one generation to the next beyond the immediate offspring. Genetic changes involve alterations in the DNA sequence that can be stably inherited across generations. Point mutations, insertions, deletions, and chromosomal rearrangements are typical examples of genetic variations that contribute to the heritable phenotypic changes observed in plants [1]. Certain genetic mutations can lead to heritable traits that persist over multiple generations, influencing traits such as flowering time, stress responses, and developmental processes [2].

Epigenetic changes, on the other hand, involve modifications to the DNA molecule or its associated proteins that affect gene expression without altering the underlying DNA sequence. These modifications include DNA methylation, histone modification, and small RNA-mediated processes [3]. Epigenetic changes are particularly intriguing because they can be reversible and responsive to environmental conditions, adding an additional layer of complexity to heritable information. Epigenetic changes can be heritable, mediating transgenerational inheritance in plants [4].

In Arabidopsis, DNA methylation has been extensively studied as a key epigenetic mechanism contributing to transgenerational inheritance. For instance, studies have demonstrated that epigenetic marks established in response to environmental stress can be maintained through meiosis and affect progeny [5].

A pivotal study by Heard and Martienssen (2014) highlighted the interplay between the genetic and epigenetic mechanisms of inheritance in Arabidopsis, revealing how epigenetic states can influence genetic stability and vice versa [6]. This interplay is crucial for understanding the full scope of transgenerational inheritance, as it emphasizes the dynamic and interconnected nature of heritable information.

Advancements in high-throughput sequencing technologies and genome-wide association studies have enabled researchers to dissect the contributions of genetic and epigenetic factors to transgenerational inheritance with unprecedented resolution. For instance, a comprehensive analysis by Iwasaki and Paszkowski (2014) elucidated how epigenetic reprogramming during gametogenesis and embryogenesis ensures the transmission of epigenetic states across generations, highlighting the robustness of these mechanisms in Arabidopsis [7].

In our previous publications, we confirmed that both genetic and epigenetic changes play crucial roles in the transgenerational inheritance of stress responses in Arabidopsis thaliana. In our studies, we evaluated changes in response to heat stress, resulting in significant physiological changes such as larger leaves and earlier bolting in the progeny of stressed plants. These changes are accompanied by reductions in global genome methylation and alterations in gene expression, including increased HSFA2 and reduced MSH2, ROS1, and SUVH genes [8]. Global genome methylation represents overall directional change in methylation in the entire plant genome. Further research demonstrated that multigenerational exposure of *Arabidopsis thaliana* (*A. thaliana* or Arabidopsis) across 25 generations to heat stress induced phenotypic resilience and increased genetic variations, with a higher frequency of homologous recombination and a greater number of mutations in stressed progeny compared to controls [9]. Similar changes were found in the progeny of Arabidopsis exposed for 25 generations to cold [10]. We also demonstrated the role of epigenetic factors, such as DNA methylation, histone modifications, small non-coding RNAs and Dicer-like (DCL) proteins, in the establishment of transgenerational inheritance in Arabidopsis [11,12].

Furthermore, we found that in response to UV-C stress, *A. thaliana* reveals both physiological and epigenetic adaptations. Exposure to UV-C for two consecutive generations resulted in a decrease in leaf number and delayed bolting in the progeny, with notable variations in seed size among different DCL mutants, where *dcl3* mutants exhibited increased seed size under stress [13]. Additionally, transposon reactivation was observed in the progeny of stressed plants, particularly for ONSEN and TSI transposons, with DCL2 and DCL3 proteins playing crucial roles in these changes. The study emphasized the importance of RNA-directed DNA methylation pathways in mediating these transgenerational responses [13].

In this study, we expanded upon our previous findings, evaluating the effect of moderate UV-C stress on *A. thaliana* plants by propagating them under UV-C for 25 consecutive generations. We demonstrate phenotypical, genomic, and epigenomic (DNA methylation) changes in the progeny of UV-C-stressed plants.

## 2. Materials and Methods

### 2.1. Parental Generation and Progeny Plants

**Figure 1:**
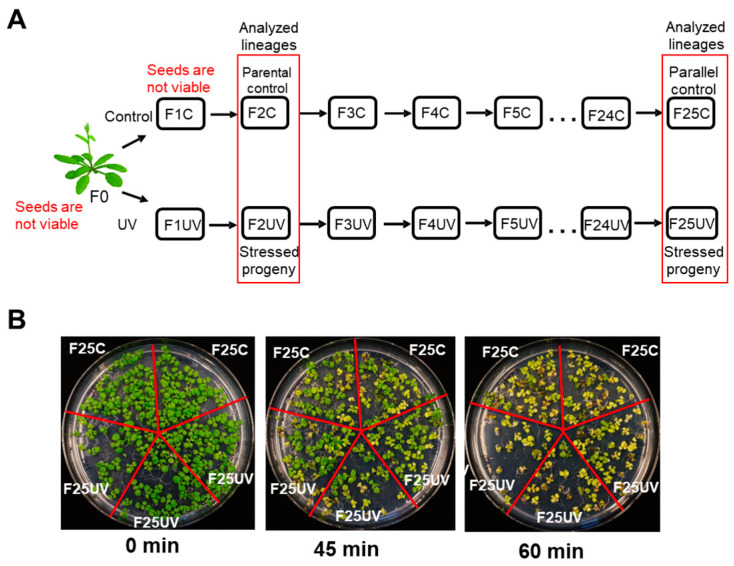
The progeny of UV-stressed plants did not show any changes in tolerance to UV stress. (**A**) Diagram of propagation of 25 generations of *Arabidopsis thaliana* (Col-0, 15d8 line): plants grown under normal conditions (C group) or exposed to UV-C (UV group). Seeds of second-generation F2C were used as parental control because seeds of F0 and F1C were no longer viable. Lineages used in this study are shown in the red box. (**B**) Phenotype of F25C and F25UV plants after 0, 45 and 60 min exposure to UV-C. Two individual control and three individual stress lineages are shown. Plants were grown on 5 × 5 cm Petri dish.

Arabidopsis seeds used as starting material representing the parental generation (F0) were obtained from a single homozygous *Arabidopsis thaliana* (Columbia ecotype, Col-0) plant transgenic for the *luciferase* (LUC) recombination reporter gene consisting of a copy of a direct repeat of the *luciferase* recombinant transgene under the control of 35S promoter. The seeds of these plants were grown in 10 × 10 cm pots, 3–4 plants per pot. Two groups of plants were obtained, those propagated without stress, the control group (C), and those propagated under UV-C stress, the stressed group (UV). Control plants were grown at normal growth conditions, with constant humidity of 65%, and with 16 h light, 8 h dark, with the following light conditions: 32.8 μEm^−2^s^−1^ of the white light at the wavelength with 2 main peaks of 540 and 610 nm. Plants representing the “UV” group were grown under the same conditions, but at the age of 7 days post germination, they were exposed to UV-C irradiation (G30T8) of 30.5 W and 99 V and UV output of 13.9 W for 2 min daily (at 10 am) for four consecutive days (Figure 1A). All parental plants were grown to flower, and seeds were collected and pooled from approximately 16 to 20 plants per tested experimental line. The seeds were stored under dry conditions at room temperature. All 25 subsequent progeny plants were obtained from these seeds; specifically, ~100 seeds were sown, and ~20 randomly selected plants formed the next generation. Five independent lineages were maintained for 25 generations. There were two lineages of control, and three lineages of stressed plants propagated in a similar manner, representing two experimental repeats of control and three experimental repeats of stressed plants.

### 2.2. Plant Growth Conditions

The seeds were kept at 4 °C for seven days to initiate stratification, and then sown in all-purpose potting soil prepared with water containing a generic fertilizer (Miracle-Gro, Scotts, Calgary, AB, Canada). The moist soil containing seeds was further stratified for 48 h at 4 °C. Plants were grown in a growth chamber (BioChambers, Winnipeg, MB, Canada) at 22 °C under the extended day conditions of 16 h of light (120 μmol photons m^−2^ s^−1^) and 8 h of darkness. Approximately three to five days post germination (dpg), plant seedlings were transplanted into individual pots containing a 9:1 ratio of soil to vermiculite composition prepared with fertilizer. Pots containing plants were placed in trays and watered from below.

### 2.3. Lineages Used for UV-Stress Phenotyping

The seeds of the F0 (parental) and F1 generations were no longer viable, hence F2C seeds were used as parental control. Overall, the lineages F2C (parental control), F25UV (the 25th generation of stressed progeny), and F25C (the 25th generation of parallel control progeny) were used for this study.

### 2.4. Phenotypic Analysis Under UV-Stress Treatment

To test the UV tolerance in the progeny, the F25UV plants were exposed to UV-C stress at 5 days post-germination, and pictures of recovery were taken a week later. Plants were exposed to UV-C irradiation (G30T8) of 30.5 W and 99 V and UV output of 13.9 for 5, 10, 20, 30, 45, 60, or 90 min. Three replicates of stressed progeny and two replicates of control progeny were used per single plate with Murashige and Skoog (MS) medium, and five sets were used for each treatment.

### 2.5. Primary Root Length Measurements

Seedlings were grown on MS-agar plates in a growth chamber at 22 °C under the extended light conditions of 16 h light and 8 h dark. At approximately 3 days after germination, the seedlings were transplanted to a new plate with MS medium to conduct the experiment and grown vertically. At approximately 5 days after germination, the experiments were conducted with the following treatments and conditions: both F25UV and F25C were irradiated with UV-C germicidal lamp located 30 cm above horizontally placed plates with no lid. Three plates containing 5 plants each were used per treatment. Depending on the treatment, full seedlings or only shoots and leaves were irradiated (roots remained covered with a white cardboard paper), either for 4 or 30 min. Plates were then kept under normal growth conditions in absence of UV-C for 4 more days. Plates were photographed before the treatment and every 24 h during the next 4 consecutive days. Primary root length was measured using the ImageJ version 1.54g software.

### 2.6. Detection of Reactive Oxygen Species

Stress treatment of plants was conducted using UV irradiation, employing the same germicidal lamp as described above. The UV-radiation source was positioned approximately 30 cm above pots containing 21-day-old Arabidopsis plants (grown under normal conditions as other treatments) from the F25UV and F25C lines. The plants were exposed to UV radiation for 30 min to induce reactive oxygen species (ROS) accumulation. At the end of the treatment, at least three leaves from five plants of both UV-stressed and control plants leaves were sampled immediately.

Hydrogen peroxide (H_2_O_2_) and superoxide anion (O_2_^−^) were used as indicators of stress due to UV exposure. For detection, 3,3′-diaminobenzidine (DAB) and nitro blue tetrazolium chloride (NBT) were used as chromogenic substrates, respectively. The staining protocol followed was as described before for DAB [14] and NBT [15]. Images of sampled leaves were analyzed using ImageJ version 1.54g.

### 2.7. Lineages Used for Genomic and Epigenomic Studies

The parental plants (F2C) and the progeny of 25th generation (F25UV and F25C) plants were grown under normal conditions for 21 days, and the rosette leaf tissues were harvested from individual plants, snap frozen in liquid nitrogen, and stored at −80 °C for DNA extraction for sequence analysis. From each generation, five individual plants from each group, F2C, F25C and F25UV, were sequenced, representing all lineages (3 from the stressed group and 2 from the control group), resulting in a total of fifteen samples for WGS and WGBS analysis.

### 2.8. Whole Genome Sequencing (WGS) and Whole Genomic Bisulfite Sequencing (WGBS)

The total genomic DNA was extracted from approximately 100 mg of leaf tissue homogenized in liquid nitrogen using a cetyltrimethylammonium bromide (CTAB) protocol. The isolated genomic DNA was used for both whole genome sequencing (WGS) and whole genome bisulfite sequencing (WGBS) to assist in identifying the genomic and epigenomic variations. The WGS and WGBS libraries’ construction and sequencing have been carried out at the Centre d’expertise et de services Génome Québec, Montreal, QC, Canada.

### 2.9. WGS Library Construction and Sequencing

For whole genome sequencing (WGS), gDNA was quantified using the Quant-iT™ PicoGreen^®^ dsDNA Assay Kit (Life Technologies, Waltham, MA, USA). Libraries were generated using the NEBNext Ultra II DNA Library Prep Kit for Illumina (New England BioLabs, Whitby, ON, Canada) as per the manufacturer’s recommendations. Adapters and PCR primers were purchased from IDT. Size selection of libraries containing the desired insert size was performed using SparQ beads (Qiagen, Venlo, The Netherlands). Libraries were quantified using the Kapa Illumina GA with Revised Primers-SYBR Fast Universal kit (Kapa Biosystems, Wilmington, MA, USA). The average size of the fragment was determined using a LabChip GX (PerkinElmer, Shelton, CT, USA) instrument.

### 2.10. WGBS Library Construction

gDNA was quantified using the Quant-iT™ PicoGreen^®^ dsDNA Assay Kit (Life Technologies). Libraries were generated with the NEBNext Ultra II DNA Library Prep Kit for Illumina (New England BioLabs) using 250 ng of genomic DNA input. Adapters were purchased from NEB. Size selection of libraries containing the desired insert size was performed using sparQ PureMag Beads (Quantabio, Beverly, MS, USA). For whole genome bisulfite sequencing (WGBS), bisulfite conversion had been carried out with the EZ DNA Methylation-Lightning Kit (Zymo Research, Irvine, CA, USA). Libraries were quantified using the Kapa Illumina GA with Revised Primers-SYBR Fast Universal Kit (Kapa Biosystems) and the average size of fragments was determined using a LabChip GX (PerkinElmer, Shelton, CT, USA) instrument.

### 2.11. WGS and WGBS Sequencing

The libraries were normalized and pooled, and then denatured in 0.05N NaOH and neutralized using HT1 buffer. ExAMP was added to the mix following the manufacturer’s instructions. The pool was loaded at 200 pM on an Illumina cBot, and the flow cell was run on a HiSeq X for 2 × 151 cycles (paired-end mode). A phiX library was used as a control and mixed with libraries at the 1% level. The Illumina control software was HCS HD 3.4.0.38, and the real-time analysis program was RTA v. 2.7.7. The program bcl2fastq2 v2.20 was then used to demultiplex samples and generate fastq reads. The bisulfite conversion rate was 99.7, and the spiked-in unmethylated lambda phage DNA was used to estimate the bisulfite conversion rate.

### 2.12. The Computation and Analysis of Genome Sequence Data

Raw sequencing reads were quality controlled and trimmed using Trim Galore software (version 0.4.4). The trimmed reads were aligned to the TAIR10 reference genome, and the duplicates were marked using the Picard tool. Local realignments around SNPs and INDELs were performed using GATK (genome analysis toolkit), which accounts for genome aligners and mapping errors and identifies the consistent regions that contain SNPs and INDELs. The resulting reads were quality controlled with Haplotype scores, and variant sample sites were called individually and jointly using the HaplotypeCaller with GATK. The sites marked as those that had a low-quality score by GATK were filtered out. The effects of variants in the genome sequences were classified using the SnpEff program [16]. According to the SnpEff program utilized in this study, upstream was defined as a 5 kb region upstream of the distal transcription start site, and downstream was defined as a 5 kb region downstream of the most distal polyA addition site. Variants affecting the non-coding regions were expounded, and biotypes were identified with the available information after comparing them with the TAIR 10 reference Arabidopsis genome.

The genomation tools were used to obtain a biological understanding of genomic intervals over pre-defined functional regions such as promoters, exons, and introns, and the Functional Classification SuperViewer was used to create gene association profiles and show the difference between samples. The genes nearest to the non-overlapping SNP and INDEL sites were annotated.

### 2.13. The Computation and Analysis of WGBS Data

Binomial tests were applied and used to determine the observed methylation frequency against the bisulfite conversion reaction, and the percentage of methylation levels was calculated at each base [17].

The WGBS raw sequencing data were analyzed using tools found in the methylKit package. Raw sequencing reads were quality controlled and trimmed using Trim Galore software (version 0.4.4). The trimmed reads were then aligned to the TAIR10 reference genome using the bisulfite mapping tool Bismark [18]. The methylated cytosines (mCs) were extracted from the aligned reads with the Bismark methylation extractor on default parameters, followed by the computation of methylation frequency using the R package software v0.5.3, methylKit. The percentage of methylation was calculated by counting the frequency ratio of Cs divided by reads with a C or a T at each base and computed at bases with coverage ≥10 [19].

$$\mathrm{The}\;\mathrm{percentage}\;(\%)\;\mathrm{of}\;\mathrm{methylation}=\left\{FrequencyofC÷readcoverage\right\}\times 100$$

Common bases covered across all samples were extracted and compared, and the differential hyper- and hypomethylated cytosines were extracted. The differentially methylated cytosines (DMCs) overlapping with genomic regions were assessed (in the preference of promotor > exon > intron), and the average percentage of methylation of DMCs around genes with the distances of DMCs to the nearest transcription start sites (TSSs) were also calculated.

The annotation analysis was performed with the genomation package within methylKit to obtain a biological understanding of genomic intervals over pre-defined functional regions such as promoters, exons, and introns [20]. The functional commentary of the generated gene methylation profiles was performed using the SuperViewer (1.0.0.2) tool with bootstrap to show the difference between samples [21]. Hierarchical clustering of samples was used to analyze similarities and detect sample outliers based on percentage methylation scores. Principal component analysis (PCA) was utilized for variations and any biologically relevant clustering of samples. Scatterplots and bar plots showing the percentage of hyper- or hypomethylated bases and heatmaps were used to visualize similarities and differences between DNA methylation profiles.

### 2.14. The Differentially Methylated Regions (DMRs)

A comparison of differential DNA methylation levels between samples reveals the locations of significant differential changes in the epigenome. The obtained information on DMRs was investigated over the predefined regions in all contexts: CG, CHG, and CHH for 100 bp and 1000 bp tiles across the genome to identify both stochastic and treatment-associated DMRs [20].

The differential hyper- or hypomethylated regions were extracted and compared across the samples. By default, DMRs were extracted with *q*-values < 0.01 and a percentage of methylation difference > 25%. The differential methylation patterns between the sample groups were also extracted. The methylation profiles of the sample groups used were F25UV versus F2C, F25UV versus F25C, and F25C versus F2C. In summary, the sliding windows of 100 bp and 1000 bp were considered for both DMRs and DMCs, and extractions were made based on at least 25% and 50% differences (*q*-values < 0.01) to assess the significant differences between samples.

### 2.15. Statistical Analysis and Quality Control Values

The mapped reads were obtained with a quality score of <30, the differential hyper- and hypomethylated bases were extracted with *q*-values < 0.01, and the percentage of methylation difference greater than 25% in methylKit. The heatmaps of differentially methylated bases were quantified at *q*-values < 0.01, and the percentage of methylation difference was more significant than 50%. The distances of DMCs to the nearest TSSs were obtained from genomation at both >25% and >50% of methylation change. The distance between TSSs and DMCs was extracted within ± 1000 bp and annotated at DMCs > 50% methylation difference. DNA methylation profiles obtained from the melthylKit used the obtained pairwise correlation coefficients of methylation levels (in %) and Pearson’s correlation coefficients for hierarchical clustering of samples. Logistic regression and Fisher’s exact test were used for the determination of differential methylation, with calculations of q-values and the Benjamini–Hochberg procedure for the correction of *p*-values. The *t*-test for the mean difference between groups was calculated with *p*-values < 0.05. The results of global genome methylation were analyzed by one-way ANOVA with Tukey’s multiple comparisons test using GraphPad Prism version 8.4.2 for Windows. The data are shown as the average percentage (with ± SD) of methylated cytosines from five individual methylomes in each of the progenies. The asterisks show a significant difference between the stressed progeny and the parental control progeny (* *p* < 0.1, ** *p* < 0.05). The phenotypic data were analyzed by the unpaired *t*-test with Welch’s correction using GraphPad Prism version 9.1.1 for Windows. The data are shown as mean ± SD. A *p*-value less than 0.05 (*p* ≤ 0.05) was considered statistically significant.

## 3. Results

### 3.1. The Progeny of UV-C-Stressed Plants Did Not Exhibit a UV-Tolerant Phenotype

To analyze the effect of UV on the progeny of *A. thaliana* plants, we propagated them on UV for 25 generations; plants grown under normal conditions served as controls (Figure 1A). To test whether F25UV plants acquired UV tolerance, we exposed them to UV for different durations of time. The experiments showed that F25UV plants were not different from F25C plants in their response to UV-C (Figure 1B and Figure S1).

### 3.2. Primary Root Elongation Is Inhibited by UV-C Irradiation, Regardless of Transgenerational Inheritance

**Figure 2:**
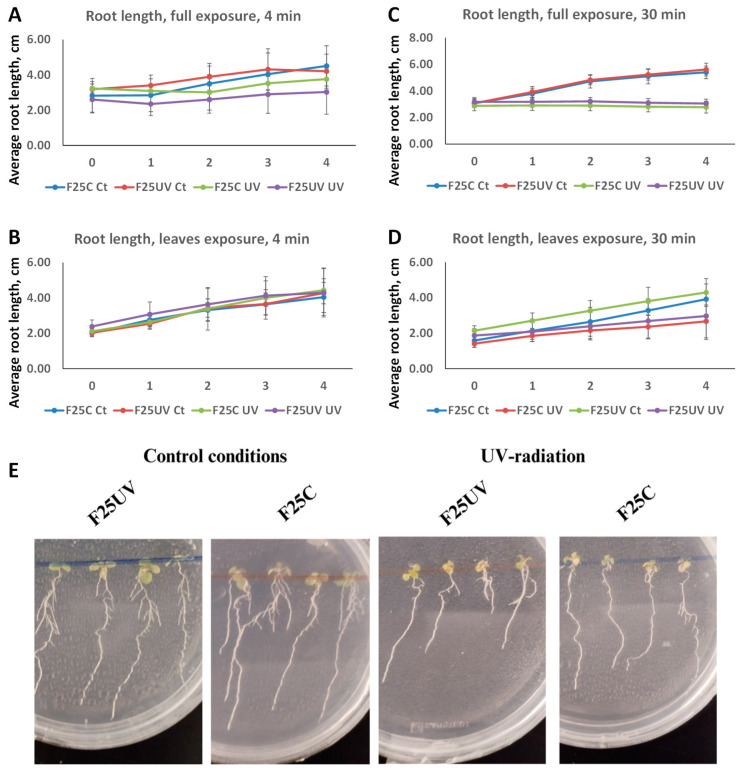
Primary root length inhibition in F25UV and F25C plants after UV-C exposure. Germicidal lamp was located approximately 30 cm above the plates (5 × 5 cm Petri dish) and measurements were taken 0–4 days after treatment. (**A**) Full seedlings were irradiated for 4 min. (**B**) Leaves and shoots were irradiated for 4 min. (**C**) Full seedlings were irradiated for 30 min. (**D**) Leaves and shoots were irradiated for 30 min. (**E**) Representative pictures of one experiment showing primary roots from seedlings of plants where leaves and shoots were exposed. In all treatments three plates containing 5 plants each were used per treatment (n = 15). Means and standard deviation are shown in the graph. Different letters indicate statistically significant differences applying Two-way ANOVA (*p* < 0.05).

To determine root growth, the F25C and F25UV generations were sown on vertically positioned plates. Whole seedlings or only their leaves were irradiated with UV-C radiation for periods of 4 or 30 min. Root length was measured before and during the subsequent 4 days (Figure 2A,B). UV-C radiation significantly reduced root growth in all cases. Plants subjected to 4 min of UV exposure showed a notable decrease in root length compared to the unexposed. Exposure of the entire plant inhibited root growth more drastically than exposure of leaves alone. However, no significant difference in root development was detected between F25C and F25UV under either control or UV treatment (*p* > 0.05). After 30 min of exposure, similar results were found (Figure 2C,D).

### 3.3. F25UV Have Similar Level of Radicals Compared to F25C Plants

DAB (3,3′-diaminobenzidine) and NBT (nitro blue tetrazolium) staining revealed distinct patterns of reactive oxygen species (ROS) accumulation in treated plants. DAB staining, which detects hydrogen peroxide (H_2_O_2_), showed localized dark brown deposits, indicating elevated H_2_O_2_ levels in specific tissues (Figure S2). In contrast, NBT staining, which detects superoxide radicals (O_2_^−^), resulted in deep blue precipitates, primarily concentrated in areas of high oxidative stress. We found no significant differences in the accumulation of free radicals in F25UV and F25C plants either grown in normal conditions or in response to UV.

### 3.4. F25UV Plants Showed a Higher Number of INDELs and Unique SNPs

**Figure 3:**
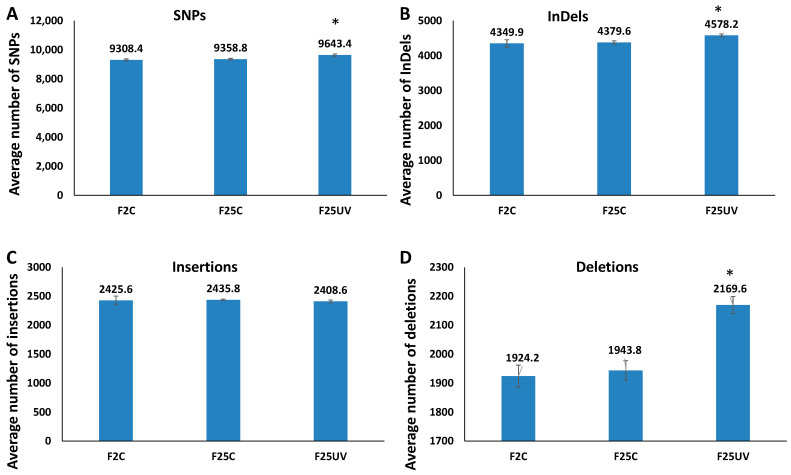
The UV-stressed progeny showed higher number of genetic variations. The total number of SNPs (**A**), INDELs (**B**), insertions (**C**) and deletions (**D**) in the genomes of stressed and control progeny and parental control when all samples were jointly considered. Y-axis shows the average (with SD), calculated from five independent biological repeats. The asterisk above (*) shows a significant difference between the F25UV and parental or parallel control generations (*t*-test, two-sample assuming unequal variances; *p* < 0.05).

To determine the frequency of occurrence of SNPs and INDELs, we mapped sequencing reads to the TAIR 10 reference genome. The total number of SNPs and INDELs was significantly higher in the F25UV group as compared to two other groups (*p* < 0.05) (Figure 3A,B). The difference in INDELs was attributed to deletions rather than insertions; there was no difference among the groups in the number of insertions (Figure 3C,D). The F25C group was not different from the F2C group.

**Figure 4:**
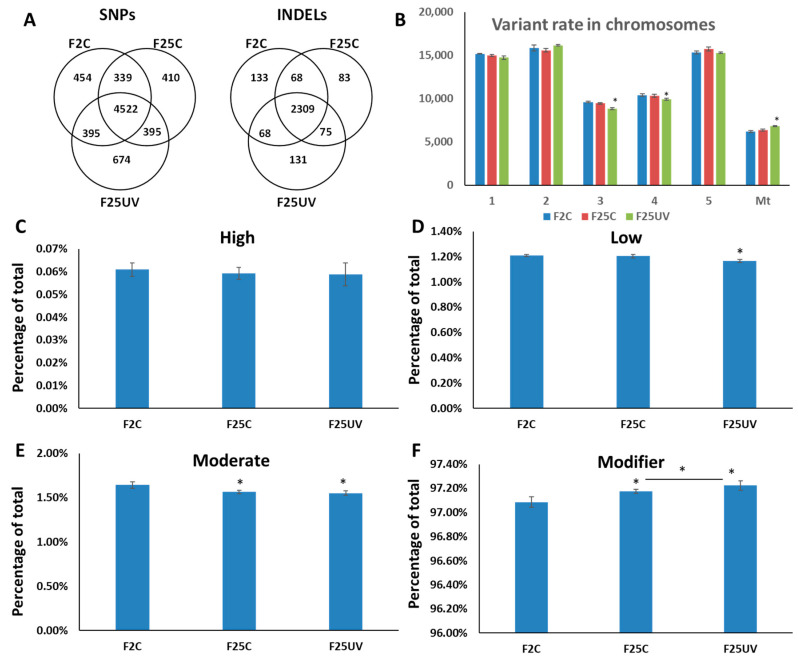
The analysis of SNP uniqueness (**A**), SNP distribution between chromosomes (**B**) and potential SNP impact (**C**–**F**). (**A**) Venn diagrams of jointly called SNP and INDEL variants. The non-overlapping area of Venn indicates the number of unique SNPs and INDELs in each sample group. Sample variants were jointly called by the “GATK haplotype caller” using five samples together. (**B**) Variant rate in different chromosomes is shown as the number of nucleotides per one SNP in each chromosome; Mt—mitochondrial genome. (**C**–**F**) Demonstrate the percentage of specific types of SNPs by their impact on gene expression, where “high” (**C**) has the highest impact on gene expression, protein composition, or/and protein function, while “low” (**D**) and “moderate” (**E**) have less of an effect. “Modifier” (**F**) has the least effect on gene expression. All terms are from SnpEff [16]. The data are averaged (with SE) from five individual plants. Asterisks over F25C or F25UV show a significant difference from FC (*t*-test, two-sample assuming unequal variances; *p* < 0.05). The asterisk over the horizontal bar indicates a significant difference between F25UV and F25C.

When unique sequence variations were considered, F25UV was found to have 674 unique SNPs accumulated, compared to 410 for F25C and 454 for F2C (Figure 4A). As far as INDELs were concerned, F25UV had 131 unique INDELs, while F25C and F2C had 83 and 133, respectively (Figure 4A).

SNPs may have different effects on the genome, from neutral to deleterious. We used SnpEff for classifying SNPs into modifiers [16], with the lowest impact on gene expression, low, moderate, and high impact, that have progressively stronger effects on gene expression (Figure 4B). There was no change in the “high” category, while there was a decrease in the “moderate” and “low” categories in F25UV compared to F2C (Figure 4C–E). Finally, there was an increase in the “modifier” category of SNPs in F25C and F25UV, which was significantly higher in the latter (Figure 4F).

**Figure 5:**
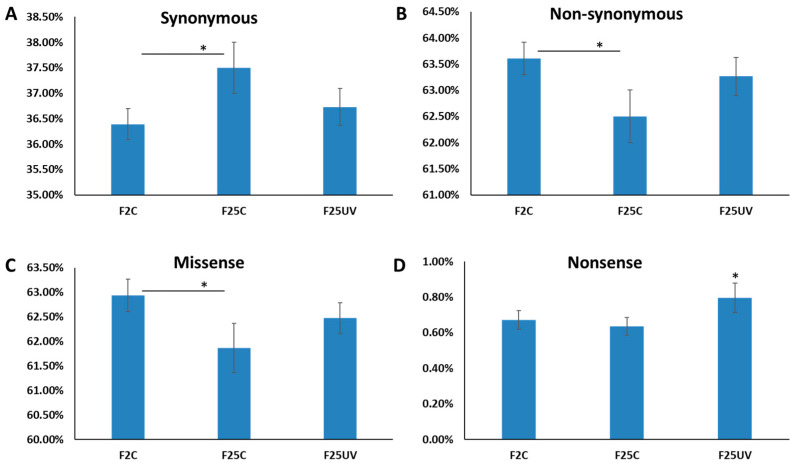
Percentage of synonymous (**A**), non-synonymous (**B**), missense (**C**), and nonsense (**D**) mutations in F2, F25C and F25UV plants. The Y-axis shows the percentage of different kinds of mutations, and the X-axis shows the plant group. Data are shown as an average ± SE, calculated from five individual samples. Asterisks over the horizontal bar show significance (*t*-test, two-sample assuming unequal variances; *p* < 0.05) between groups, while asterisk over F25UV group shows the difference between this group and either F2C or F25C, *p* < 0.05.

We also analyzed SNPs in categories of synonymous and non-synonymous mutations, with further categorization of non-synonymous mutations into missense and nonsense mutations. There was a significantly higher percentage of synonymous and a significantly lower percentage of non-synonymous mutations in F25C as compared to F2C (*p* < 0.05) (Figure 5A,B). The F25C group had a lower percentage of missense mutations as compared to the F2C group, while the F25UV group had a significantly higher percentage of nonsense mutations than either the F2C or F25C groups (Figure 5C,D).

**Figure 6:**
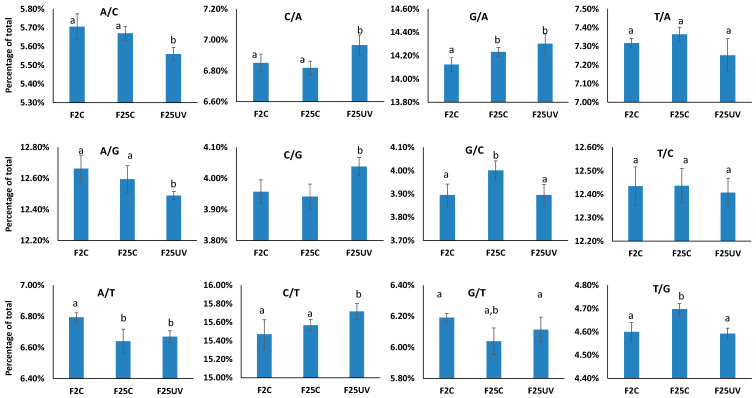
Type of single nucleotide substitutions in F2, F25C and F25UV groups. The Y-axis demonstrates the percentage of mutations among all mutations, while the X-axis shows the three tested groups. Data are shown as an average from five samples ± SE. Letters (a,b) show significant differences (*t*-test, two-sample assuming unequal variances; *p* < 0.05). A—adenine, C—cytosine, G—guanine, T—thymine.

Point mutations in the form of nucleotide changes are not equally distributed; some mutations are more frequent than others. We analyzed mutations by nucleotide change and found C to T and G to A mutations to be the most frequent among all other mutations (Figure 6). The F25UV group also had a significantly (*p* < 0.05) higher percentage of C to G and C to A mutations than either the F25C or F2C groups (Figure 6). The F25UV group also had a lower percentage of A to C and A to G mutations (*p* < 0.05) (Figure 6). The most common mutation type was the C to T transition. Also, the percentage of all mutations at cytosines (C/T, C/A and C/G) was significantly higher in the F25UV group. The analysis of mutations of cytosines in the CpG context or at other cytosine locations (CHG and CHH contexts) showed that there was no significant difference between the percentage of C/T, C/A, or C/G mutations among F2C, F25C, or F25UV (Figure S3). It can thus be suggested that a higher frequency of mutations at cytosines occurs at CHG and CHH sites.

SNPs are often unevenly distributed in the genome. We only found a significant difference among the groups in the intergenic regions: a higher percentage of SNPs was in F25UV as compared to other groups (Figure S4).

**Figure 7:**
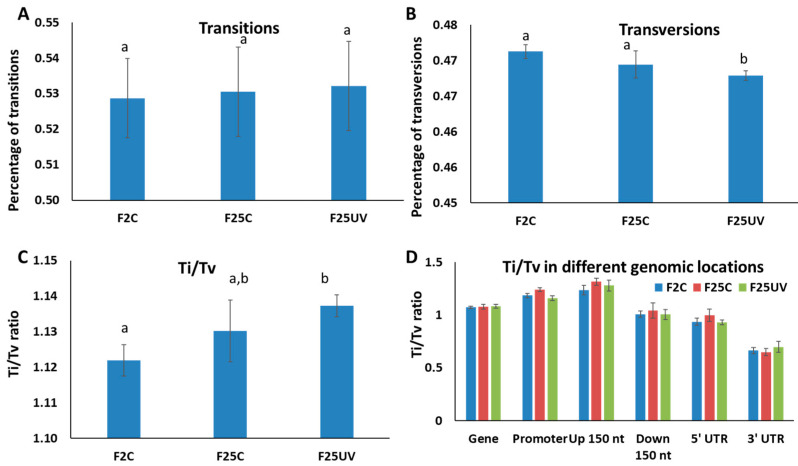
Transitions and transversions in the F2C, F25C and F25UV groups. Percentage of transitions (**A**), transversions (**B**) and the ratio of transition to transversion (Ti/Tv) (**C**) as well as the ratio of Ti/Tv in various genic regions (**D**). The Y-axis shows either percentage of Ti or Tv (**A**,**B**) or the Ti/Tv ratios (**C**,**D**), while the X-axis shows the specific group. Data are shown as an average calculated from five different samples, with SE. Letters over the bars show the significant difference (*t*-test, two-sample assuming unequal variances; *p* < 0.05) between the groups.

Mutations of purine to purine and pyrimidine to pyrimidine are called transitions (Ti), while purine to pyrimidine and pyrimidine to purine are called transversions (Tv). Ti mutations are commonly more frequent than Tv in most organisms, and there is also evidence that the Ti/Tv ratio changes in response to stress. We found the rate of transitions was similar among all three groups (Figure 7A), while the rate of transversions was significantly (*p* < 0.05) lower in the F25UV group than in the other two groups (Figure 7B). We also found that the Ti/Tv ratio was significantly higher in the F25UV and F25C groups as compared to the F2C group (Figure 7C); even though the Ti/Tv ratio was larger in the F25UV as compared to the F25C group, the difference was not significant. We found no significant difference in the Ti/Tv ratios in different genomic locations among groups (Figure 7D).

### 3.5. Genetic Variants in F25UV Were Enriched in Several Cellular Components, Molecular Functions and Biological Processes

**Figure 8:**
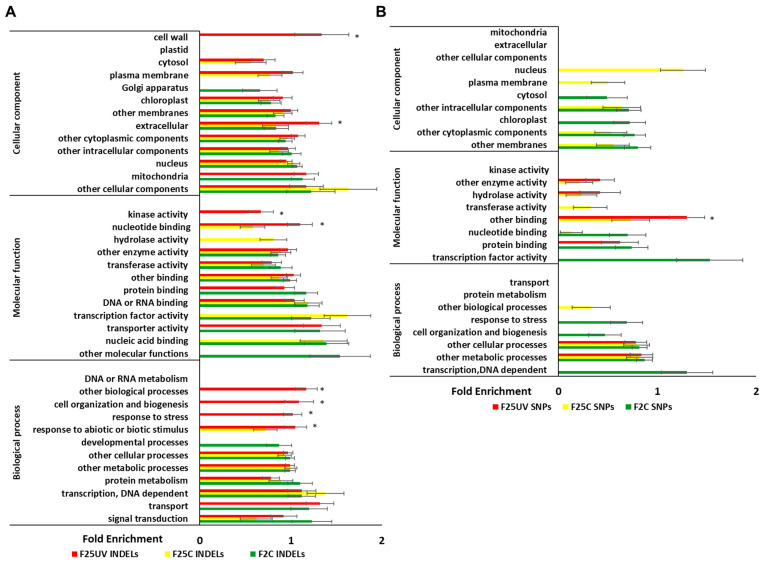
Enrichment of cellular components, molecular function, and biological processes in INDELS (**A**) and SNPs (**B**) in F2C, F25C and F25UV groups. The X-axis shows normalized class score with binomial coefficients as calculated by SuperViewer. To calculate enrichment, *p*-value of <0.05, ±bootstrap SD were used, significance is shown with asterisk. The Y-axis shows cellular components, molecular functions, and biological processes.

Enrichment analysis of INDELs revealed that the cellular components “cell wall” and “extracellular” were significantly enriched in the F25UV group, but not in the other groups (Figure 8A). Molecular functions “kinase activity” and “nucleotide binding” and biological processes “cell organization and biogenesis”, “response to stress”, “response to abiotic and biotic stimulus”, and “other biological processes” were also enriched in F25UV as compared to other groups. In SNPs, the molecular function “other binding” was enriched in the F25UV group (Figure 8B).

### 3.6. Multigenerational Exposure to UV-C Increases Global Genome Methylation at the CpG Sites

**Figure 9:**
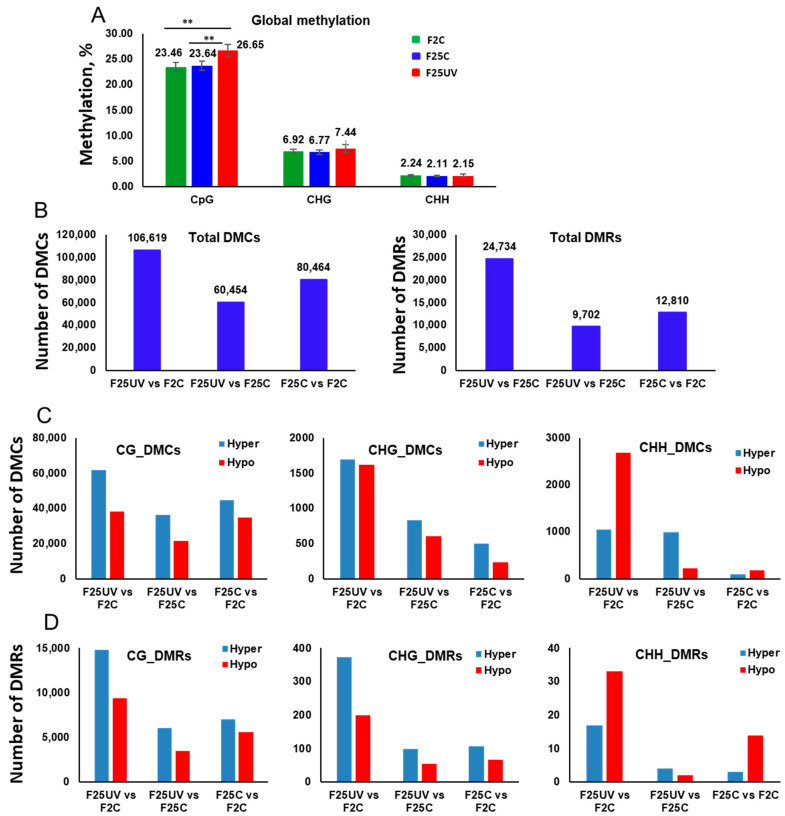
Global methylation changes, differentially methylated cytosines (DMCs) and differentially methylated regions (DMRs) in the F25UV, F25C and F2C plants. (**A**) The average percentage of methylated cytosines in F2C, F25C, and F25UV in CG, CHG, and CHH sequence contexts (H = A, T, C). Methylation levels were determined from reads with the minimum coverage ≥ 10 mapped to the TAIR 10 reference genome by using Bismark software (version 0.24.2). The data were analyzed by one-way ANOVA with the Tukey’s multiple comparisons test using GraphPad Prism version 8.4.2 for Windows. The data are shown as the average percentage (with ±SD) of methylated cytosines from five individual methylomes in each of the progenies. The asterisks show a significant difference between the stressed progeny and the parental control progeny (** p < 0.05). (**B**) The total number of DMCs and DMRs in the F25UV vs. F2C, F25UV vs. F25C and F25C vs. F2C comparison groups. (**C**) The total number of hyper- and hypomethylated DMCs in the CG, CHG and CHH contexts in the F25UV vs. F2C, F25H vs. F25C and F25C vs. F2C comparison groups. (**D**) The total number of DMRs in CG, CHG and CHH contexts in the F25UV vs. F2C, F25UV vs. F25C and F25C vs. F2C comparison groups.

Analysis of DNA methylation showed that global genome methylation was significantly higher in the F25UV plants as compared to the F25C and F2C plants in the CG context, while it was similar at the CHG and CHH sites, although there was a trend toward increased methylation at the CHG sites (Figure 9A).

### 3.7. The F25UV Group Shows a Higher Number of DMCs and DMRs in Comparison to the F2C Group

Analysis of DMCs showed that F25UV plants had more differentially methylated cytosines than the F2C group at all contexts; they also had more DMCs in comparison to the F25C group at CHG and CHH contexts, while the F25C group in comparison to the F2C group had the lowest number of DMCs (Figure 9C). The most striking difference was observed for CHH context, where F25UV comparison to F2C revealed over 10-fold more DMCs at CHH position as compared to the control group comparison of F25C to F2C.

Our data suggest that in terms of methylation, the F25UV group is substantially more divergent from the F2C group, than the F25C group; at the same time, the F25UV group is more similar to the F25C group than to the F2C group. Furthermore, our data suggest that multigenerational UV-C stress resulted in the increased number of hypermethylated cytosines at CG sites; there was an equally increased number of hyper- and hypomethylated cytosines at CHG sites, and a drastically increased number of differentially methylated cytosines at CHH sites, with hypomethylated cytosines prevailing. Analysis of DMRs revealed a very similar picture, except that at symmetrical CG and CHG contexts, the F25UV group was different from the F2C group, predominantly due to hypermethylated cytosines (Figure 9D).

### 3.8. Plants Belonging to the F25UV and F2C Groups Cluster Separately in All Cytosine Contexts

**Figure 10:**
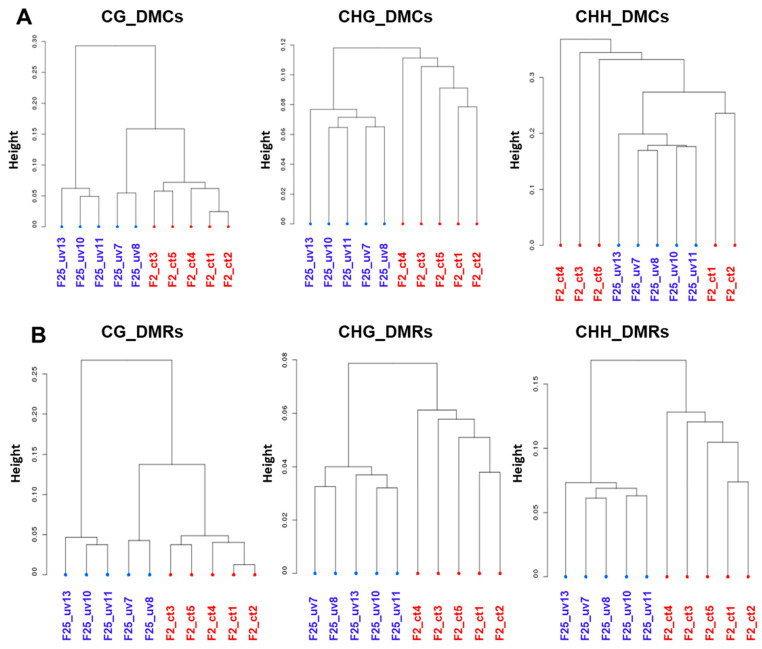
Global methylation clustering of DMCs (**A**) and DMRs (**B**) at CG, CHG and CHH context in F25UV vs. F2 comparison. Hierarchical clustering of F25UV vs. F2C using Pearson’s correlation distance. “Height” indicates the distance of split.

To analyze whether individual plants in the F25UV and F2C groups are different from each other, we performed cluster analysis. For DMCs, F25UV and F2C clustered completely separately at symmetrical CG and CHG, while at non-symmetrical CHH, the F25UV plants clustered together, while the F2C plants were split into two groups (Figure 10A). For DMRs, the F25UV and F2C groups clustered separately in all sequence contexts (Figure 10B). Clustering of the F25UV and F25C groups did not reveal complete separation in the CG and CHG context but showed it in the CHH context (Figure S5). Finally, clustering of F25C and F2C demonstrated rather random positioning of individual samples (Figure S6).

**Figure 11:**
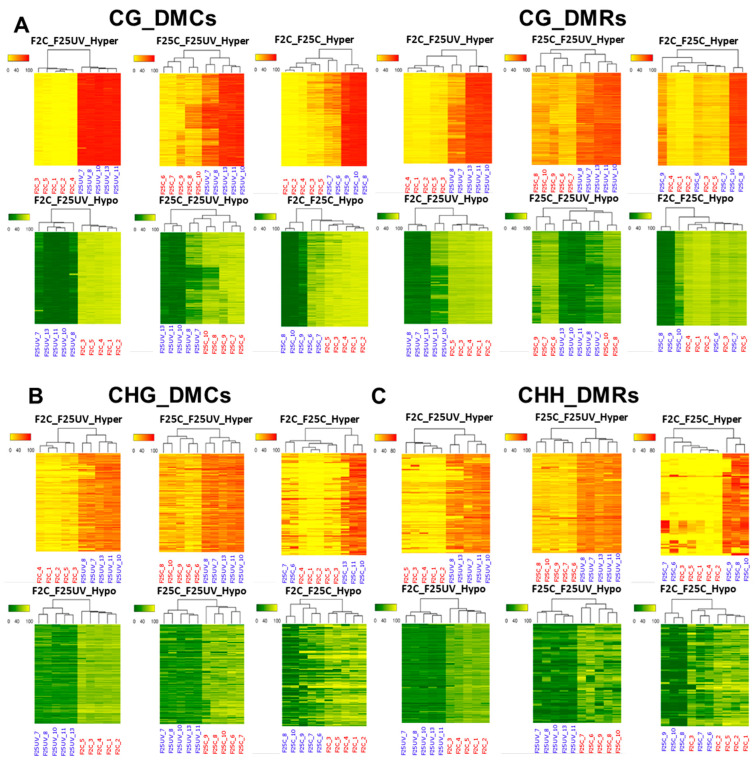
A hierarchical clustering heatmap analysis. Heat maps of DMCs and DMRs for hypermethylated cytosines (the upper panel) and hypomethylated cytosines (the lower panel) in the CG (**A**), CHG (**B**) and CHH (**C**) contexts in the F25UV vs. F2C, F25UV vs. F25C and F25C vs. F2C comparison groups. Differentially methylated cytosines in the genome with differences of >50% in F25H vs. F2C. In the upper panel, the red section indicates the larger percentage of methylation, and the yellow section indicates the lower percentage, and in the lower panel, the green section indicates the larger percentage of methylation, and the yellow one indicates the lower percentage, *q*-value < 0.01.

We continued our analysis of the relatedness of samples using the hierarchical clustering heatmap analysis (Figure 11). The heatmap analysis of DMCs in all three contexts revealed a clear separation of five samples of the F25UV group from five samples of the F2C group or samples of the F25C groups, while comparison of F25C to F2C had less clear separation, with several F25C samples clustering together with F2C samples (Figure 11). A similar picture was observed for DMRs in the CG context. This analysis revealed that while the F25UV group was different from the F2C and F25C groups, F25C and F2C appeared to be similar.

### 3.9. Analysis of Methylation in the Genic and Intergenic Regions

**Figure 12:**
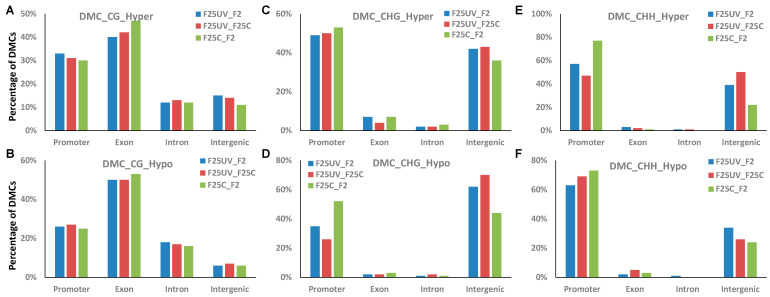
The distribution of hyper-and hypomethylated DMCs in the genic and intergenic regions in F25UV vs. F2C, F25UV vs. F25C and F25C vs. F2C comparison groups. (**A**) Distribution of hypermethylated CG DMCs. (**B**) Distribution of hypomethylated CG DMCs. (**C**) Distribution hypermethylated CHG DMCs. (**D**) Distribution hypomethylated CHG DMCs. (**E**) Distribution of hypermethylated CHH DMCs. (**F**) Distribution of hypomethylated CHH DMCs.

DMCs and DMRs were then characterized related to their genic and intergenic position. The location of hypo- or hypermethylated DMCs and DMRs was compared to the annotated Arabidopsis genes using the genomation Bioconductor package [19]. Both hyper- and hypomethylated DMCs at the CG context were predominantly located in the genic regions, with a higher fraction found in the gene body, specifically in the exons (Figure 12A). No substantial differences between comparison groups were found; however, we noticed that the F25UV group in comparison to F2C or F25C groups had a lower percentage of hypermethylated DMCs in the exon region and a higher percentage in the promoter region as compared to the F25C vs. F2C group comparison (Figure 12A).

When similar analysis was performed in the CHG and CHH contexts, we found a large fraction of DMCs in the promoter or intergenic regions. For hypomethylated CHG, a drastic difference was found for DMCs in the intergenic regions; there were over ~20% more DMCs in the F25UV comparison groups as compared to the F25C vs. F2C comparison groups. The opposite was observed for promoter regions—F25UV comparison groups had a lower percentage of hypomethylated DMCs as compared to F25C vs. F2C group (Figure 12D). In the CHH context, there was the decrease in the hypermethylated and hypomethylated cytosines in the promoter region in the F25UV as compared to F25C and F2C groups, and the increase in the intergenic region (Figure 12E).

In DMRs, no substantial differences were observed in the CG context (Figure S7). In the CHG context, changes in DMRs were like those in DMCs; over 20% more hypomethylated DMRs were found in the F25UV comparison groups as compared to the F25C vs. F2C comparison group in the intergenic region, while the opposite was observed in the promoter regions (Figure S7D). In the CHH context, there was an increase in the hypermethylated cytosines in the promoter region in the F25UV as compared to F25C and F2C groups, and the decrease in the intergenic region (Figure S7E). The opposite was found for hypomethylated DMRs in the CHH context: a decrease in the promoter regions and an increase in the intergenic regions (Figure S7F).

### 3.10. Pathway Enrichment for Epimutation-Associated Genes

**Figure 13:**
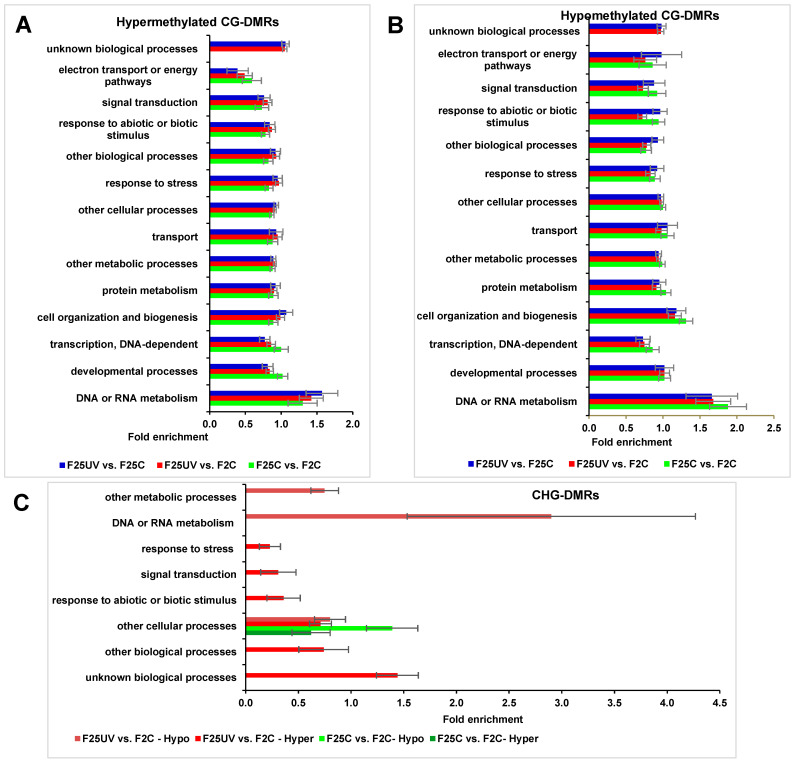
The enrichment analysis of DMRs in the CG and CHG sites and their classification based on biological processes. (**A**). Hypermethylated DMRs in CG context. (**B**). Hypomethylated DMRs in CG context. (**C**). Hyper- and hypomethylated DMRs in CHG context. The X-axis shows normalized class score with binomial coefficients as calculated by SuperViewer. To calculate enrichment, *p*-value of <0.05, ±bootstrap SD were used.

Gene Ontology (GO) analysis of DMRs in the hyper- and hypomethylated CG contexts did not show any substantial differences in the enrichment of biological processes; “unknown biological processes” were enriched in the F25UV comparison groups but were absent in the F25C vs. F2C group (Figure 13A,B). There were differences in “DNA and RNA metabolism”, but they were not significant. In contrast, when the same was analyzed in the CHG context, we found an enrichment in the biological process “DNA or RNA metabolism” in the F25UV hypomethylated group, and under-representation in “response to stress”, “response to biotic and abiotic stimulus”, “signal transduction” and “other biological processes” in the F25UV hypermethylated group (Figure 13C).

## 4. Discussion

In this work, we demonstrated that exposure of plants to UV-C for 25 generations results in a significant increase in mutations and epimutations but does not result in changes in tolerance to UV-C as compared to the progeny of plants propagated under normal conditions.

### 4.1. Absence of Phenotypic Resilience to UV-C Stress

To evaluate the resistance of F25UV plants to UV-C, several different experiments were performed. We first tested visual differences in the appearance of UV-irradiated F25C and F25UV plants and did not observe any differences (Figure 1B). We also propagate these plants in soil to maturity and did not observe any differences between two lineages. Then, we irradiated plants grown in vitro, by either exposing whole plants or leaves only, and measured the roots; this time, we performed quantitative analysis. Still, we did not observe any differences in root growth/length between F25C and F25UV plants. Finally, we measured the level of oxidative stress and free radicals in these plants, and again, saw no differences. The absence of significant differences between the F25C and F25UV plants suggests the absence of phenotypic resilience to UV-C.

Our previous research identified transgenerational phenotypic changes induced by the UV-C radiation, such as decreased leaf number in the progeny of UV-C-stressed plants, alterations in bolting time, and larger seed size observed in the F1 progeny of stressed plants [13]. These findings suggest that it is feasible to expect further development of phenotypic resilience with continuous exposure to UV-C. It is possible that such an adaptive response to UV-C specifically is transient in nature since UV-C is not a stress that plants are commonly exposed to. It could be that such continuous exposure results in an adaptive response that is not distinguishable from the response of naïve plants; indeed, we did not observe any difference in the response of the F25UV and F25C plants to UV-C. While previous studies showed that UV-B repressed primary root elongation [22], results of Zhang et al. (2024) were similar to ours; rice plants were subjected to UV-C for four generations; analysis of G1 and G2 showed that plants exhibited a significant decrease in the grain length, grain width, spike weight, and thousand-grain weight, along with an increase in empty grain percentage and proanthocyanidin content compared to natural light conditions, demonstrating a negative response to UV-C [23]. However, in generation G3, these parameters returned to control levels. This supports our observations; in our previous studies we noted phenotypic changes in the first and second generations in response to heat [8], UV-C [13], and heavy metals [24]. In this study, UV-C-exposed plants did not differ phenotypically from control plants in F25 generation.

In contrast, analysis of the progeny of plants exposed to heat for 25 generations showed that F25H (the progeny of heat-exposed plants) were more resilient to heat stress as compared to F25C (the progeny of control plants) [9]. The divergence in outcomes might be attributed to differences in the type of stress. Since UV-C is not naturally present on the Earth’s surface, no specific receptor for this type of radiation or responsive mechanism have been found in plants so far. Consequently, the stress induced by UV-C may lack significant biological relevance for plants in the short term from an evolutionary perspective.

Some studies have suggested that transgenerational effects may not be a general response to abiotic stress in Arabidopsis [25]. This indicates that the transmission of stress memory through epigenetic mechanisms may not always result in increased adaptability to stressors in plants and could be influenced by specific conditions or genetic factors. As seen in other cases, recurring biotic stress can induce transgenerational inheritance, although it does not necessarily imply the adaptability of an organism to a particular condition [26,27]. In another study, Arabidopsis was subjected to mild drought in a multi-generational experiment. Although plastic responses were observed, the descendants of stressed and non-stressed plants were phenotypically indistinguishable after being propagated without stress, regardless of whether they were grown under control conditions or water deficit [28].

### 4.2. The Analysis of Genetic Variations Induced by Multigenerational Exposure to UV-C

UV radiation can damage DNA directly and can lead to the increase in mutation rates in plants. Many other stresses, including X-ray and gamma-rays, as well as various chemicals can also damage the DNA [29]. In contrast, many common stresses, such as changes in temperature, water availability and even pathogen exposures can trigger increase in mutation rate, likely via increase in production of reactive oxygen species [30,31,32,33]. The progeny of plants exposed to stress also exhibit an increase in mutation rate and homologous recombination frequency [9,11,33,34,35,36,37].

UV-C induces two major types of photoproducts, including cyclobutane-type pyrimidine dimers (CPDs) and pyrimidine (6-4) pyrimidone photoproducts (6-4 PPs). These dimers, when not properly repaired lead to a variety of single nucleotide and tandem mutations. Exposure of Arabidopsis plants to 500 and 1000 J/m^2^ of UV-C resulted in 26- and 45-fold increased mutation frequency, respectively [38]. G:C to A:T transitions were predominant, representing over 50% of all mutations. Curiously, when UV-C was followed by photoreactivation with white light, the frequency of these mutations would dramatically decrease, indicating efficient photoreactivation repair. The predominant type of mutations in such photoreactivated plants, ~56%, was A:T to T:A transversions [38].

In our current work, C to T transitions and G to A transitions were also predominant types of mutations that have increased in the progeny of UV-C-exposed plants (Figure 6). In fact, the mutations at cytosines (C/T, C/A and C/G) were all significantly higher in the F25UV group. Similarly, our analysis of the progeny of cold-stressed plants showed that these mutations also prevail [10].

The increased mutability of 5-methylcytosines has influenced patterns of genetic diversity and genomic evolution in many different species. CpG dinucleotide occurrence is much rarer than randomly predicted and more likely than other dinucleotides to exhibit polymorphism [39]. Additionally, C to T mutations are disproportionately prevalent among variants linked to both diseases [40] and adaptation to new environments [41,42]. As discussed by Pértille et al. (2019), CpG mutation rates in natural populations could be influenced by environmental exposures affecting their methylation status [43]. In addition to influencing point mutations, DNA methylation at CpG sites also contributes to the formation of larger genomic rearrangements. They play a key role in regulating transposable element activity, which can lead to the creation of new genomic structures, including insertions, deletions, and duplications [44].

Another example of large genomics changes is the increase in the frequency of somatic homologous recombination observed in the progeny of Arabidopsis plants treated with short-wavelength UV-C radiation or flagellin, a bacterial elicitor [37].

### 4.3. The Analysis of Epigenetic Variations Induced by Multigenerational Exposure to UV-C

Analysis of DMCs showed that F25UV plants had more differentially methylated cytosines than the F2C group in all sequence contexts; it also had more DMCs in comparison to F25C groups at CHG and CHH contexts. The difference in the CHH context was the most dramatic; over 10-fold more cytosines were differentially methylated at the CHH position in the F25UV group as compared to either control group. Similar differences were observed in the progeny of plants exposed to cold for 25 generations; an over 4-fold increase in the number of DMCs was observed in these plants as compared to control plants, with the increase in hypomethylated DMCs being over 10-fold [10]. Also, an approximately 2-fold increase in DMCs at the CHH position was observed in the progeny of plants exposed to heat for 25 generations [9]. These findings echo those of Wibowo et al. (2016), who noted stress-induced CHH methylation changes in plants, although they, too, did not observe direct resilience traits arising from these epimutations [45].

Studies have shown that the exposure of Arabidopsis to stressors like UV-C radiation and bacterial elicitors can induce heritable changes in gene expression and chromatin modifications [46,47]. Furthermore, studies have demonstrated that UV exposure can lead to epigenetic effects in plants, such as changes in DNA methylation, which can influence plant defence systems and responses to stress even further [48]. Such epigenetic alterations can be maintained for several generations [49,50].

In a recent study, Laanen et al. (2021) utilized a multigenerational setup in which three generations (Parent, Generation 1, and Generation 2) of 7-day-old Arabidopsis thaliana plants were exposed to different gamma radiation levels for 14 days [51]. They found that the greatest number of changes occurred at CG, with fewer changes observed in CHG methylation and none in CHH methylation. Generation 2 showed a substantially higher number of DMRs than the Parent and Generation 1. Surprisingly, plants exposed to the highest dose rate showed the fewest differences. They hypothesized that this could indicate that a certain threshold was crossed at which the plants switch to a different method of coping with the IR exposure. Probably a similar response may occur in UV-C radiation at different intensity levels.

This transgenerational memory, observed in plants like Arabidopsis, involves mechanisms such as DNA methylation, histone modifications, and small RNA pathways, which play crucial roles in transmitting stress responses across generations [52,53].

We found that hyper- and hypomethylated DMCs in the CG context were predominantly located in the genic regions, with a higher fraction found in the gene body, and specifically in the exons, a phenomenon called gene body methylation (gbM). However, no substantial differences between comparison groups were found, although we noticed that the F25UV group, in comparison to the F2C or F25C groups, had a lower percentage of hypermethylated DMCs in the exon region and a higher percentage in the promoter region. Both CHG and CHH genic methylation types are associated with reduced expression levels, as is CG methylation in promoter regions [54]. However, the exons of some genes (~20% of *A. thaliana* genes) are methylated only in the CG context [55]. This type of methylation that occurs specifically in the CG context, positively correlates with gene expression, and is enriched over constitutively expressed genes, mostly moderately and constitutively expressed housekeeping genes [56]. However, as reviewed by Muyle et al. (2022), evolutionary and comparative studies based on genetic diversity or species comparisons have frequently, though not universally, shown a link between gbM and gene expression, particularly in relation to expression stability [57]. This observed association between gbM and expression is noteworthy as it may represent a phenotype that natural selection could act upon. Furthermore, it has been found that transgenerational inheritance of acquired gbM helps environmental canalization of gene expression, facilitating long-term stress adaptation of mangroves facing of a severe reduction in genetic diversity. Indeed, gbM may allow rapid adaptation to novel environments, since gbM epimutation rates are nearly 10^5^ times higher than genetic mutation rates [58,59].

In our study, we observed a substantially larger number of epigenetic as compared to genetic changes. While, on average, there were ~14,000 genetic changes in each group, including SNPs and INDELs, there were 60,000 to over 100,000 differences in methylation, depending on comparison group. The F25UV group only had a couple hundred more SNPs and INDELs than either control group, while for differentially methylated cytosines, this group was different from F2C by over 100,000 cytosines. This suggests that while the genome of plants exposed to UV-C stays relatively stable over 25 generations of exposure, the epigenome “evolves” significantly. Previous research shows that there are more variations in the epigenome rather than in the genome of Arabidopsis grown under normal conditions [60,61]. This is what we observed here and in previous reports. The data on the comparison of genomic and epigenomics changes in response to stress in the same plant on the level of whole genome are scarce. Data we found demonstrated that exposure to salt [62], heat [9] and cold [10] stresses, leads to more drastic changes in the genome rather than in the epigenome in the progeny. Again, it is very likely that diversification in the epigenome is a feature of a response to an uncommon stress, such as UV-C, while exposure to more familiar stressors, like temperature and salinity may not require robust epigenetic responses.

While we found substantial variation in the epigenome of F25UV plants compared to F2C plants, the analysis of the pathways potentially altered by these changes did not show any drastic differences. The biological process “DNA or RNA metabolism” was enriched in F25UV plants, reflecting changes in the genome (mutations) and epigenome (changes in methylation could be due to the differential activity of non-coding RNAs and in turn lead to differential transcriptome). The F25UV group had an under-representation of biological processes “response to stress”, “response to biotic and abiotic stimulus”, “signal transduction” and “other biological processes” in the hypermethylated group. This suggests that there were fever genes that were hypermethylated that belonged to these processes. In turn, it means that these processes were likely more active in the F25UV group, as compared to control groups, and that they would likely contribute to the better ability of F25UV plants to withstand stress.

The results of our study revealed that multigenerational UV-C exposure in Arabidopsis thaliana induced significant genetic and epigenetic changes but did not result in increased UV tolerance, challenging previous assumptions about the potential adaptive value of such mutations. Despite the accumulation of unique SNPs and DMCs, no phenotypic resilience to UV stress was observed in the F25UV generation. Like our findings, earlier studies demonstrated that stress-induced epigenetic modifications do not always confer direct fitness advantages [45]. For example, Ferreira et al. (2015) observed no improved salt tolerance in multigenerational stressed rice despite an increase in epigenetic variability [63]. This supports the hypothesis that epigenetic changes might play roles beyond immediate adaptive responses, potentially contributing to evolutionary processes at a longer timescale.

## 5. Conclusions

UV radiation was shown to induce priming in plants, enhancing the resistance to other types of stress, especially drought and salinity [64,65]. In this context, evaluating stress lines under various conditions could enhance our understanding of the transgenerational plasticity of this germline. Nevertheless, our study has several limitations that warrant consideration. Primarily, we focused on the 25th generation of seeds produced under UV and non-UV conditions, rather than evaluating each generation. This approach may overlook incremental adaptive changes that could occur over successive generations. Also, we did not find solid evidence of phenotypic changes in the progeny of plants exposed to UV-C for 25 generations. In several instances, we noted slightly higher tolerance of F25UV plants upon exposure to UV-C; we likely must increase the number of plants under study and add other analyzed physiological parameters. Also, analyzing the response to other stresses may show more prominent differences. Additionally, evaluating programmed cell death and DNA damage responses would provide a more comprehensive understanding of the mechanisms underlying root growth inhibition under UV stress. It would also be interesting to analyze methylation in a tissue-specific manner, to identify any tissue-specific transgenerational response.

## Data Availability

Original sequencing data were deposited at https://www.ncbi.nlm.nih.gov/bioproject/PRJNA802915, accessed on 15 December 2022.

## Supplementary Materials

The following supporting information can be downloaded at: https://www.mdpi.com/article/10.3390/life15030502/s1, Figure S1. UV stress phenotype at mature stage; Figure S2. Representative images of leaves from each treatment group; Figure S3. Analysis of point mutations at cytosines; Figure S4. Genomic location of single nucleotide substitutions in F2, F25C and F25UV groups; Figure S5. Hierarchical clustering of DMCs (A) and DMRs (B) at CG, CHG and CHH context in F25UV vs. F25C comparison using 1-Pearson’s correlation distance; Figure S6. Hierarchical clustering of DMCs (A) and DMRs (B) at CG, CHG and CHH context in F25UV vs. F2 comparison using 1-Pearson’s correlation distance; Figure S7. The distribution of hyper-and hypomethylated DMCs in the genic and intergenic regions in F25UV vs. F2C, F25UV vs. F25C and F25C vs. F2C comparison groups.

## Abbreviations

The following abbreviations are used in this manuscript:

DMC	Differentially methylated cytosine
DMR	Differentially methylated region
UV-C	Ultraviolet C radiation
SNPs	Single nucleotide polymorphisms
INDELs	Insertions and deletions
CG	Methylation at cytosine followed by guanine
CHG	Methylation at cytosine followed by any nucleotide except guanine, followed by guanine
CHH	Methylation at any cytosine
DCL	Dicer-like protein
WGS	Whole genome sequencing
WGBS	Whole genome bisulfite sequencing
TSS	Transcription start site
ROS	Reactive oxygen species
F2C	Second generation of progeny of control plants
F25C	Twenty-fifth generation of progeny of control plants
F25UV	Twenty-fifth generation of UV-stressed plants
Tv	Transversions
Ti	Transitions
