# Supplementary material for: Genetic and Epigenetic Changes in Arabidopsis thaliana Exposed to Ultraviolet-C Radiation Stress for 25 Generations
Source: Life (Basel). 2025 Mar 20;15(3):502. doi: 10.3390/life15030502 (PMC11943796; doi:10.3390/life15030502)
Supplement: Supplementary file 1 [file life-15-00502-s001.zip › life-3433005-supplementary.pdf]

SUPPLEMENTARY MATERIAL

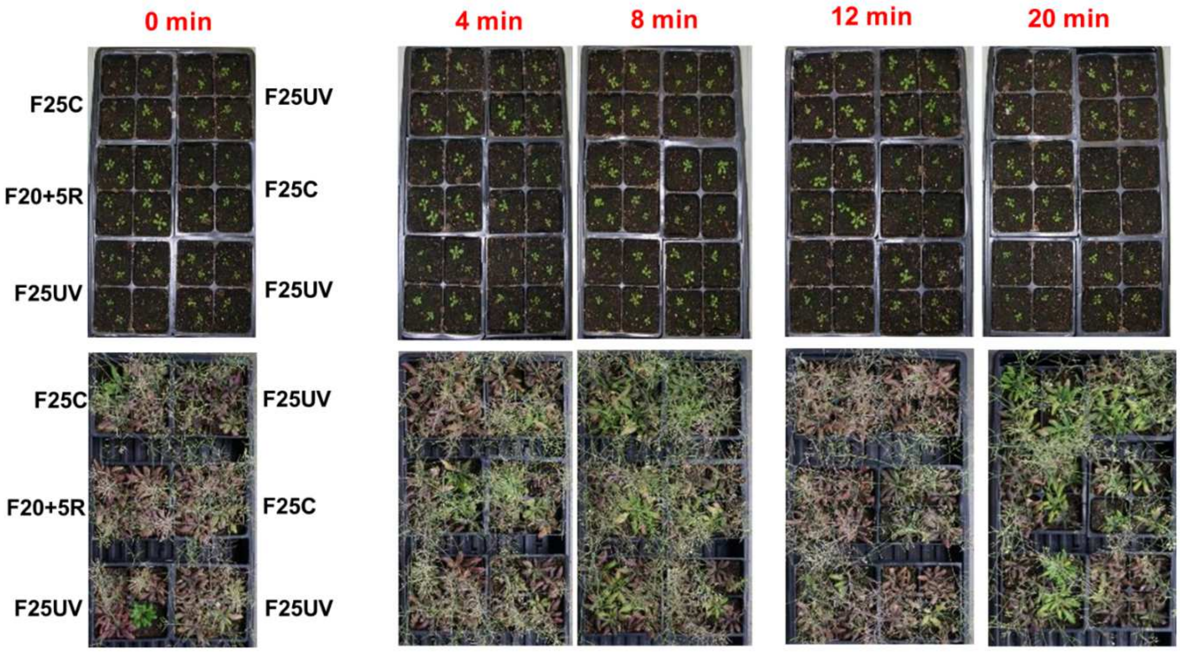

Figure S1. UV Stress phenotype at mature stage. UV stress progeny (F25UV) did not show any difference in growth as compared to parallel control progeny (F25C) under UV stress. An additional F20UV after 5 more generations without stress were included. UV stress was given at 10-days old seedlings. Growth conditions were the same as in the other experiments. Recovery photograph after 11-days of UV treatment.

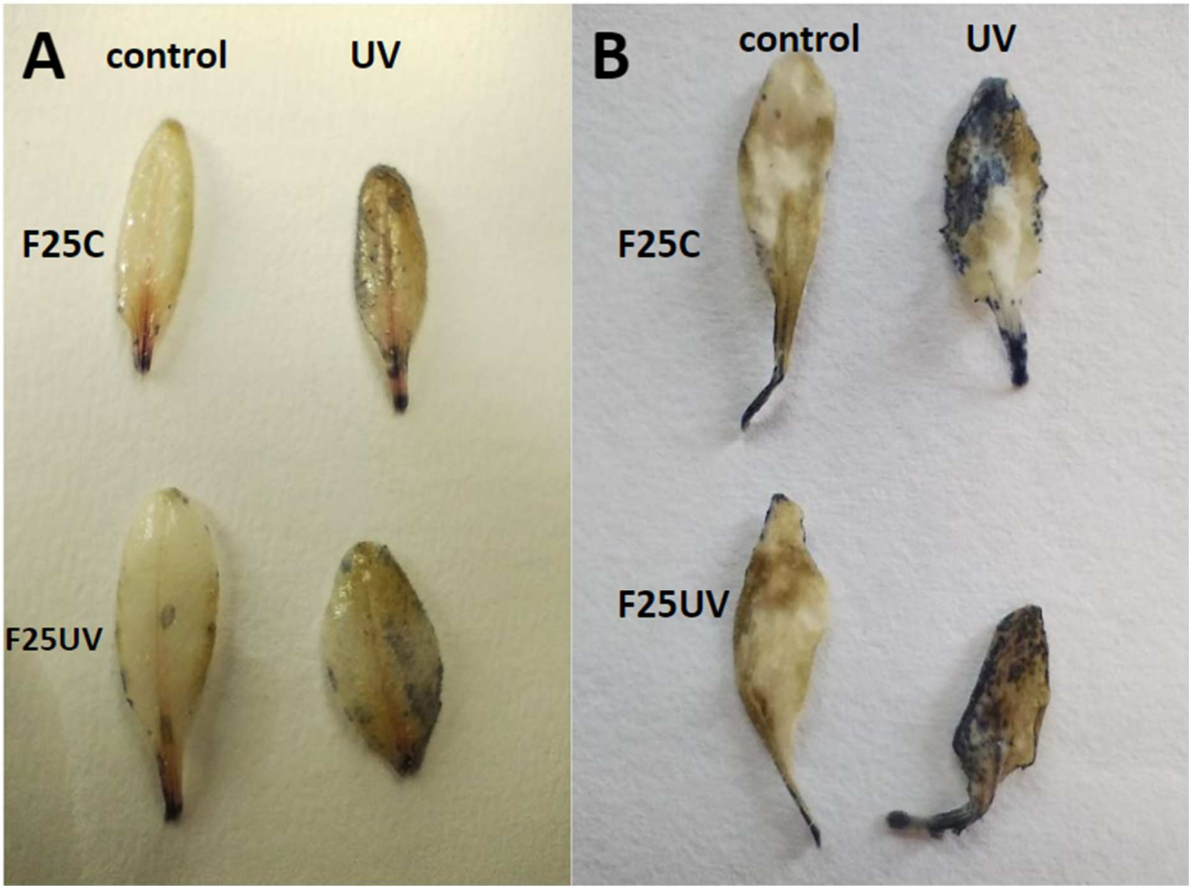

Figure S2. Representative images of leaves from each treatment group. Leaves from DAB (A) and NBT (B) protocol assessment. Treatments showed belong to control plants and plants exposed to UV radiation. The figure shows the most representative leaves for each treatment, from a total of at least 3 leaves each from 5 different plants.

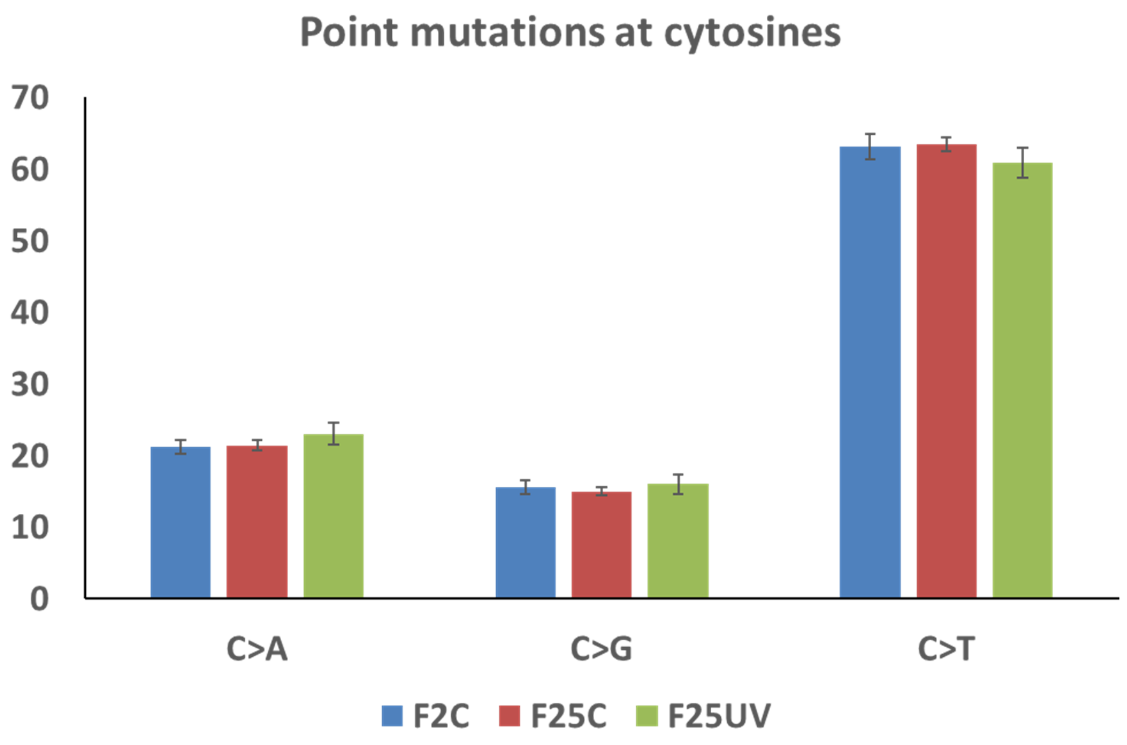

Figure S3. Analysis of point mutations at cytosines. Y axis shows the percentage of total. Bars show average with standard error calculated from 5 samples.

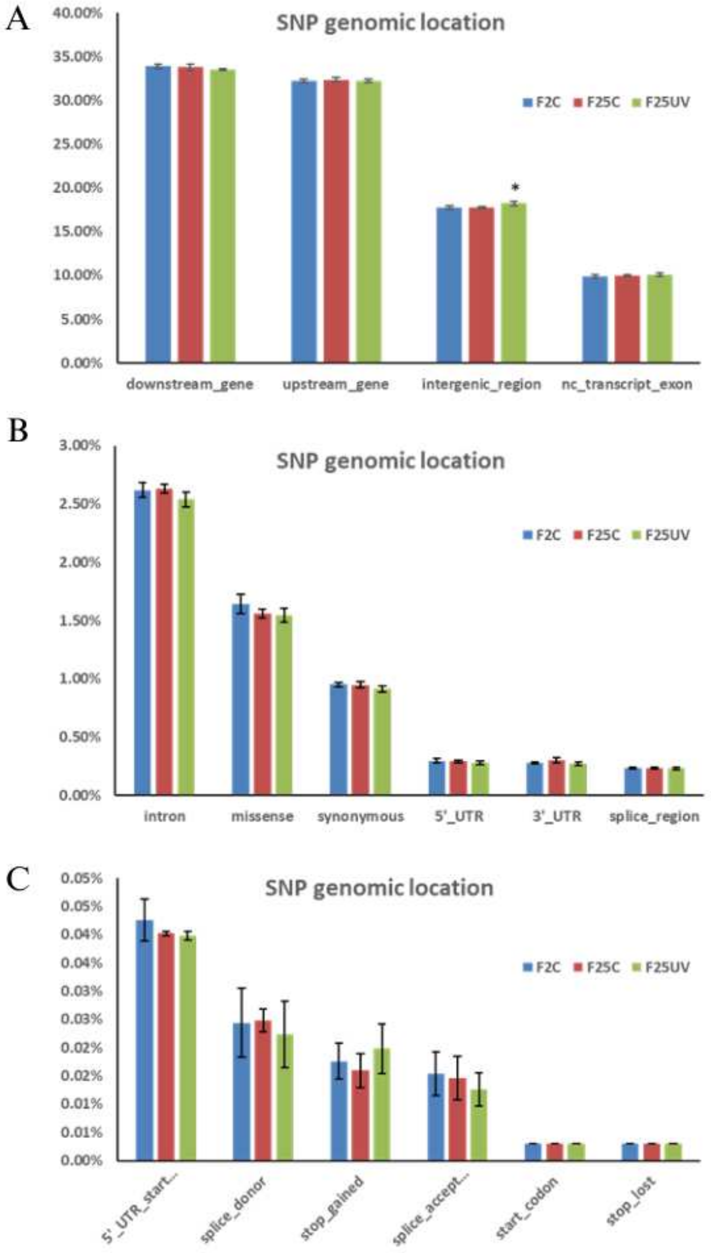

Figure S4. Genomic location of single nucleotide substitutions in F2, F25C and F25UV groups. A, B and C show different genomic locations according to the frequency of occurrence. Y axis demonstrates the percentage of mutations among all mutations, while X axis shows the genomic location groups. Data are shown as an average from five samples, with SE. Asterisk shows significant differences (t-test, two-sample assuming unequal variances; $p<0.05$).

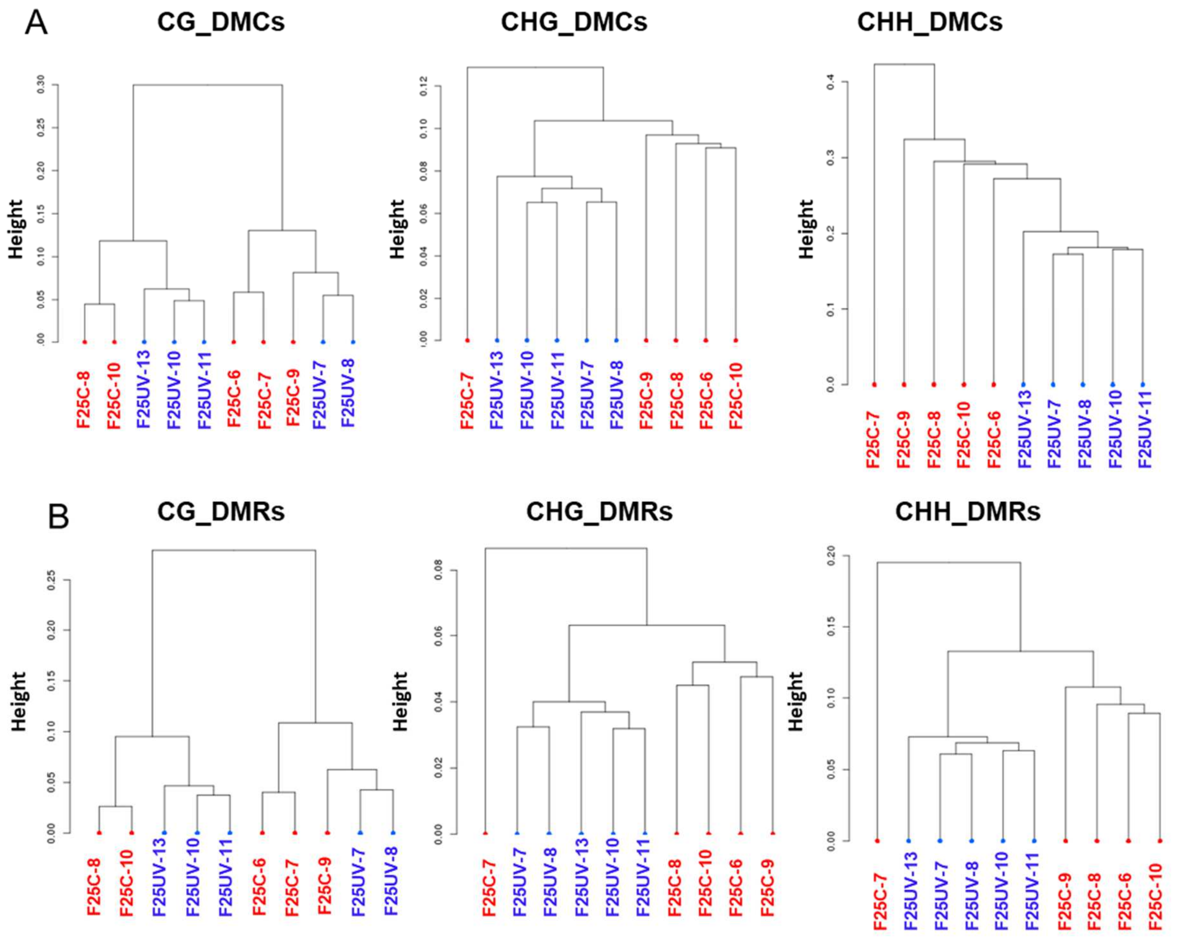

Figure S5. Hierarchical clustering of DMCs (A) and DMRs (B) at CG, CHG and CHH context in F25UV vs F25C comparison using 1-Pearson's correlation distance. "Height" indicates the distance of split.

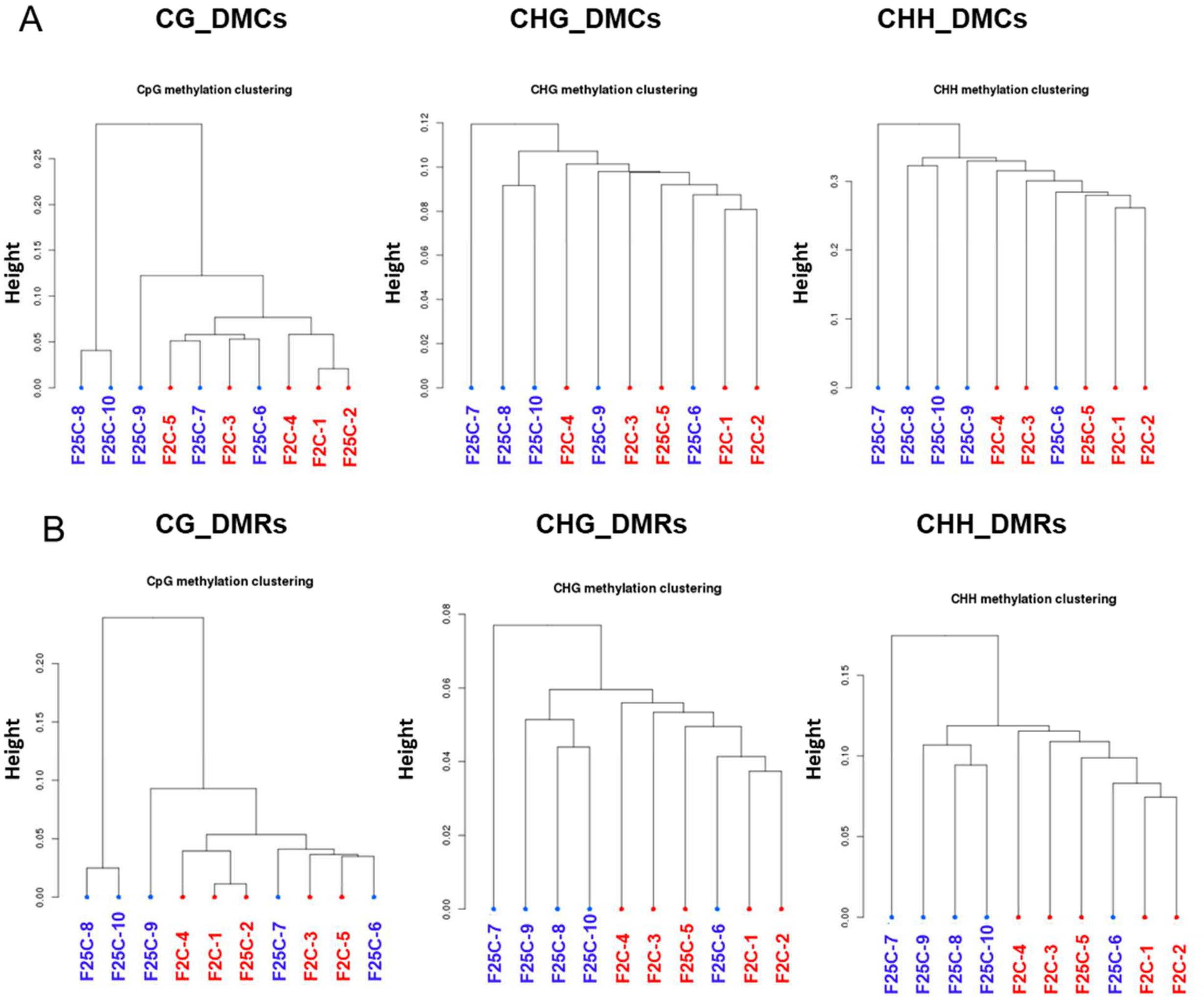

Figure S6. Hierarchical clustering of DMCs (A) and DMRs (B) at CG, CHG and CHH context in F25UV vs F2 comparison using 1-Pearson's correlation distance. "Height" indicates the distance of split.

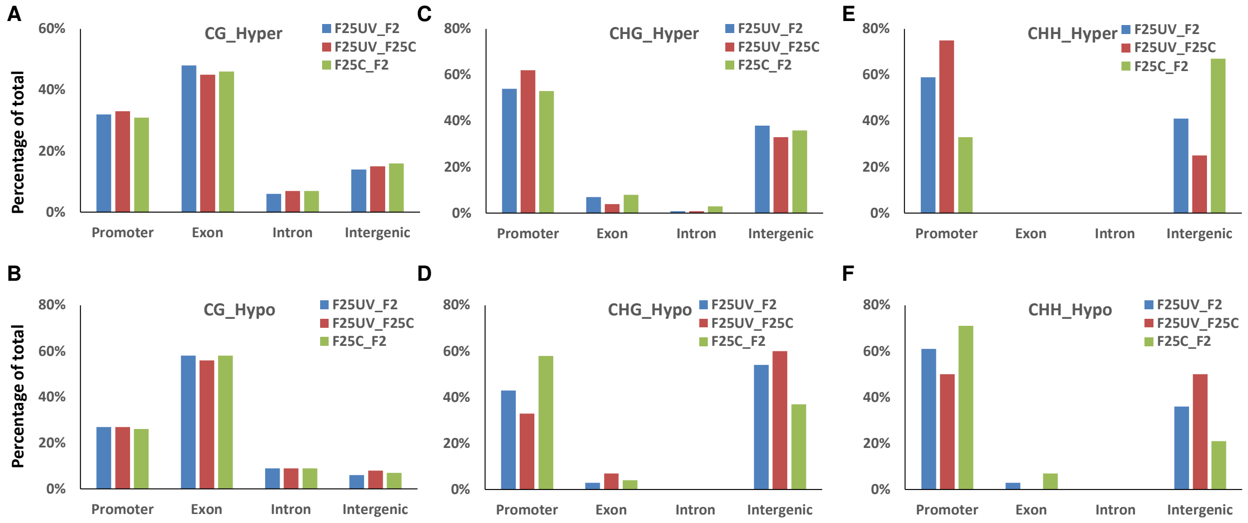

Figure S7. The distribution of hyper-and hypo-methylated DMCs in the genic and intergenic regions in F25UV vs. F2C, F25UV vs F25C and F25C vs. F2C comparison groups. (A) Distribution of CG DMCs and DMRs. (B) Distribution of CHG DMCs and DMRs. (C) Distribution of CHH DMCs and DMRs.
